# Organizational Framework Conditions for Workplace Health Management in Different Settings of Nursing—A Cross-Sectional Analysis in Germany

**DOI:** 10.3390/ijerph19063693

**Published:** 2022-03-20

**Authors:** Hannah Bleier, Jasmin Lützerath, Andrea Schaller

**Affiliations:** 1 Working Group Physical Activity-Related Prevention Research, Institute of Movement Therapy and Movement-Oriented Prevention and Rehabilitation, German Sport University Cologne, 50933 Cologne, Germany; j.luetzerath@dshs-koeln.de (J.L.); a.schaller@dshs-koeln.de (A.S.); 2 Research & Development Team, Institute for Workplace Health Promotion, Neumarkt 35–37, 50667 Cologne, Germany

**Keywords:** workplace health management, workplace health promotion, nurses, care facilities, organizational framework conditions, cross-sectional analysis, Germany

## Abstract

Studies show that workplace health promotion (WHP) can reduce sickness-related absenteeism among employees and secure long-term workability. Embedding WHP in workplace health management (WHM) can contribute to sustainability and holism. This study aimed to investigate organizational framework conditions for WHM in different settings of nursing in Germany (acute care hospital, long-term care (LTC) facilities and home-based LTC). In a project on WHM implementation, managers with personnel responsibility for nurses (*n* = 16) were surveyed. In total, 46 close-ended questions on organizational framework conditions of WHM in their care facility were answered at the beginning of the project. No significant differences were found for the indexes of *health promoting willingness*, *health promoting management*, *social capital* and *workplace health activity*. Descriptive analysis showed that home-based LTC performed slightly better on average. Home-based LTC and LTC facilities had higher ratings in *health promoting willingness* than in actually managing the process (*health promoting management)*, while the results for acute care hospitals were reversed. Acute care hospitals showed the lowest values for the topics of health as a leadership topic and evaluation of incidents of violence, which were generally rated lower among all settings. Need for action can be identified in improving personal, financial and time resources, evaluation and information on WHM.

## 1. Introduction

At the latest from the time of the coronavirus pandemic, the working conditions in nursing have become the focus of public attention. As nursing has come to be regarded as systemically important, the health burden on nursing staff has also become the focus of political and public attention, especially since the number of sick days in this occupational group has been above average for years [1,2].

In addition to the current pandemic, the system relevance of this occupational group results from the constantly growing number of old and multi-morbid people due to the demographic development. At the same time, there are signs of a lack in nurses in Germany [2]. Given the demographic trend of people in need of care currently, in 2030 there will have to be one new nurse for every 4.5 nurses to meet the demand [2]. If the course is not set soon for more personnel in nursing, Germany will have to deal with considerable supply shortages within a few years [2]. In addition to the projected shortage of nurses, the health care industry also faces several burdens in the day-to-day workplace. There is an increased risk of long-term absence due to illness and early retirement [3]. The most common diseases diagnosed by doctors are musculoskeletal disorders and mental health problems. Compared with other professions (19.3 sick days), nurses had an average of six more sick days (25.2) in 2020. Indeed, this tendency has been quite stable since 2015 [4]. Besides the actual number of sick days, nurses also show significantly lower working capacity compared with other occupational groups in Germany [5]. An impact on health and well-being might also be due to violence emanating from patients. In a cross-sectional study among German care facilities, 94.1% mentioned having experienced verbal abuse and 69.8% had experienced physical aggression in the previous 12 months [6]. As a result of violent incidents, nurses reported becoming more careful, vigilant, and tense, as well as having less enjoyment in their work and in interactions with patients, clients and residents [6].

Adding to the sick days, the problem of fluctuation and the high workload to which nurses are exposed, the need for action becomes clear [7].

Studies show that workplace health promotion can reduce employee absenteeism due to illness and secure employees’ ability to work in the long term [8]. A look at the German history of work-related health promotion shows that the topic was already associated with corporate success in the 19th century. In occupational health and safety, the first working time regulations were issued in 1829 and the first accident prevention regulations in 1984 [9]. From this more health-protective view, workplace health promotion then began to be anchored in management processes in the 1980s. This went beyond the original health protection [9]. Today WHM in Germany comprises all health-related activities focusing on maintaining and promoting health and it can be seen as the roof of three columns: legally required occupational safety, legally required *operational integration management* (OIM) and the voluntary workplace health promotion (WHP) [9,10].

Behavioral-related and environmental-related workplace health promotion programs are an acknowledged approach to counteract health-related challenges. For example, for increasing physical activity, quitting smoking and promoting healthy diets, there are small to moderate positive impacts for health across all sectors [10]. Embedded in workplace health management (WHM), they have been proven to lead to an increase in quality and a sustainable anchoring of the topic of health in companies [11]. This way, both employers and employees can benefit from this [12].

A review of nursing-specific intervention studies reveals a range of effectiveness evidence. Studies examining holistic approaches to health promotion show good results in terms of health, stress and workability. In an RCT study, nurses were able to select stress management measures at the organizational and personal levels [13]. In the intervention group, a reduction in presenteeism as well as absenteeism and higher productivity were evident [14]. A program developed for ambulatory caregivers (COMPASS) that focused on improving community spirit, education about health promotion, occupational safety, and healthy eating and fitness opportunities, showed significant improvements in health status and reductions in participants’ experience of stress in a pre/post comparison after six months [15]. The design of a break room and its close location led to an improvement in the quality of meal breaks and a significant reduction in the stress levels of nurses [16]. Improved working conditions and increased staffing in inpatient care were found to reduce stress [17]. Practical insights show a wide range of health promoting company activities in care facilities [7]. Nevertheless, in German care institutions often only superficial or solely behavioral-related activities for health are implemented, while a holistic and systematic approach is missing [7]. Consequently, there is potential regarding environmental-related health promotion [7].

Organizational framework conditions supporting this holistic approach are considered to have unused potential in WHM [18]. For the further development of WHM, existing structures should be used or appropriate new structures should be established to systematically manage WHM [19]. The importance of structures and processes as organizational framework conditions to continuously ensure holistic WHM is also underpinned by the Federal Institute for Occupational Safety and Health in Germany, which has defined promotion factors for occupational safety and workplace health. Thereby, a structured WHM plan, process monitoring and evaluation are recommended for building up a WHM process [20,21]. In addition to the formation of structures, the provision of adequate resources in terms of personnel and finances is considered important [22,23]. Employee participation [11] and the formation of steering groups or similar recurring meetings is also described as a success factor [23,24]. Furthermore, the networking of internal and external actors is proven to be an important criterion for success in the implementation of a holistic approach to WHP in hospitals [14]. Implementing a holistic WHM needs inter-agency cooperation [25] and the systematic integration of WHP activities, OIM and occupational safety [19].

Against this background, WHM structures and procedures hold strong importance in maintaining job attraction, workforce and health of the nurses. Up to now, however, there has been no setting comparison of organizational framework conditions in the field of nursing. Based on this, the present study addresses the following research question: Which differences in WHM structures and processes can be seen in different settings of nursing (acute care hospital, long-term care (LTC) facilities and home-based LTC)?

To answer this question, it was decided to measure the structures and processes of five main topics, which can provide a picture of the organizational framework conditions:

− health promoting capacity;
− social capital;
− workplace health promotion (as the first pillar of WHM);
− *operational integration management* (as the second pillar of the WHM);
− occupational safety (as the third pillar of WHM);
− dealing with violence and aggression (as a special burden in nursing).

## 2. Materials and Methods

The present study is part of the “BAGGer” project (Workplace offers for health promotion and violence prevention in WHM: impact model-based conception and evaluation of a WHP programme) funded by the Federal Ministry of Health (BMG) and registered in the German Clinical Trials Register (DRKS-ID: DRKS00024961). The project started in November 2020, ends October 2022, and is approved by the ethics committee of the German Sport University Cologne (reference numbers No. 050/2021). Briefly summarized, a target group-specific WHP program for the participating care facilities (acute care hospital, LTC facilities and home-based LTC) is participatively developed and implemented as one column of WHM within the BAGGer project. The implemented WHP programs are evaluated based on an impact model.

### 2.1. Study Design

A cross-sectional data analysis of the organizational structures of 16 care facilities was conducted, among which five facilities were home-based LTC, seven LTC facilities and four acute care hospitals (see Table 1). For the underlying project mentioned in the previous paragraph, the participating institutions were recruited through convenient sampling.

For the present study, the following inclusion criteria were defined at the person level: (a) knowledge about structures of WHM and (b) to have far-reaching personnel responsibility for nursing employees in the surveyed care facility. The final sample therefore comprised directors of nursing in acute care hospitals or care managers in LTC facilities and home-based LTC. The sample was obtained by sending an information sheet about the survey to participating care institutions within the BAGGer project. The information sheet included brief information about the purpose of the study, the duration of the questionnaire (30 min) and the guarantee of anonymity. All participants provided informed consent. The data collection period was from March to June 2021. The questions on WHM structures and processes (see below in the instruments section) and the related response options were read out to the participants and the respective responses were documented in an editable PDF file by the researchers (J.L. and H.B.). The data collection was conducted by telephone.

### 2.2. Instrument for the Operationalization of WHM Structures and WHM Processes (WHM Check)

To measure structures and processes of the organizational framework conditions for WHM, a compiled instrument, the WHM-Check, was used. The WHM check comprised different questionnaires. Overall, the WHM check contained 46 closed-ended questions divided into six main topics about WHM structures and processes. The main topics were health promotion capacity [26,27] comprising *health promoting willingness* (mapping the will of a company to implement WHM on a permanent basis) [26,28] and *health promoting management* (the extent to which WHM is being put into practice systematically in the form of a management process) [26,28]. *Social capital* [28] was measured (the scope of a network and the capital that exists in social relationships among the employees in the different settings [29]), as well as *workplace health promotion* [28], *handling of incidents of violence and aggression* [30], *operational integration management* [31] and *occupational safety*. The questionnaires used to assess the six main topics are listed in Table 2. Detailed overview of the questionnaires and items used for the WHM-check can be found in the supplementary material (Table S1).

### 2.3. Statistical Analysis

For describing *health promoting willingness* in every setting, we decided to build an index (0 = “do not agree at all”, 10 = “fully agree”) over three items by averaging. In the same way, there was an index formed for every setting by averaging nine items to describe *health promotion management* [28]. In the next step, we used these index results and compared them with established cut-off values from the same dimensions in the *Worksite Health Promotion Capacity Instrument (WHPCI)* to classify the facilities in their degree of *health promoting capacity.* Hereby, the cut-off for *health promoting willingness* was >5.9 and for *health promoting management >2.6* [26,27]. According to this, we checked which of our care facilities showed results above both cut-off-marks to attest them a “high” *health promoting capacity* [26,27].

Averaging the 9 items of *social capital* enabled calculating a *social capital* index for every setting (0 = “do not agree at all”, 10 = “fully agree”) [28]. For *workplace health activities*, there was an index calculated (0 = “do not agree at all”, 10 = “fully agree”) in the same way by taking the average of six items on behavior-related and environmental-related activities aiming to promote health and displaying them per setting [28].

For the indexes of *health promoting willingness, health promoting management, workplace health activities* and *social capital*, the mean (mean), median (med), standard deviation (±SD), frequency (n) and percentage (%) were calculated per setting as descriptive analyses. To examine the setting-specific differences, a univariate analysis of variance was calculated. For *workplace health structures* [28], *handling of incidents of violence and aggression* [30], *operational integration management* [31] and *occupational safety*, frequencies (n) and percentages (%) for every item were reported. To ensure a conservative approach to the interpretation of the results (avoidance of an alpha error) and due to the rather small sample, we decided to set the significance level at *p* < 0.01. All statistical analyses were run with IBM SPSS 26 (IBM Corp., Armonk, NY, USA).

## 3. Results

Regarding the willingness of a company to implement WHM on a permanent basis (*health promoting willingness index*), home-based LTC (7.4 ± 1.2) showed the highest score, while the acute care hospital setting (5.9 ± 1.2) showed the lowest score. The extent to which WHM is being put into practice systematically in the form of a management process (*health promoting management index)* was the highest for the setting of acute care hospitals (6.3 ± 2.0) and lowest for LTC facilities (5.5 ± 2.9). Comparing the two dimensions, home-based LTC and LTC facilities have higher ratings in *health promoting willingness* than in actually working on a WHM management process (*health promoting management index)*. For hospitals, the results were the opposite, as the *health promoting willingness* was lower than the actual WHM process (*health promoting management index)*.

The degree of *health promoting capacity* depending on *health promoting management* and *health promoting willingness*, showed that 100% of the home-based LTC, 86% of the LTC facilities and 75% of the acute care hospitals achieved a *“high” health promoting capacity*. The *social capital*—an index displaying the quality of social relationships—was best rated for home-based LTC (3.3 ± 0.2) and lowest rated for acute care hospital (2.6 ± 0.3).

Although acute care hospitals showed the best *health promoting management index* (WHM being put into practice systematically in the form of a management process), the index for *workplace health activities* examining behavioral-related and environmental-related activities was the lowest over all settings (5.1 ± 1.0). The highest results for the *workplace health activities index* were achieved in the setting of home-based LTC (6.3 ± 2.6), which also had the best results for the *health promoting willingness.*

Table 3 shows the median, minimum, maximum, variance and mean of the *health promoting willingness index, health promoting management index, social capital index* and *workplace health activity index*. Univariate analysis of variance showed no statistically significant difference in the indexes according to the different settings.

**Figure 1:**
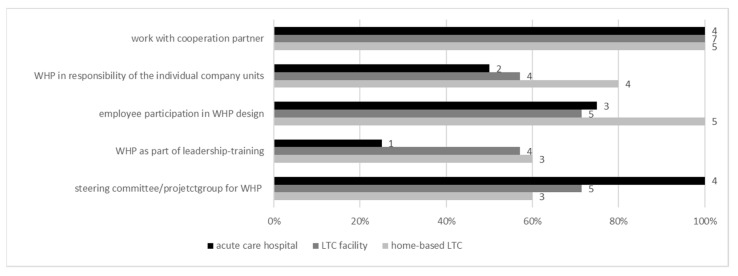
Existence of *workplace health structures* (per setting).

Examining the *workplace health structures* that provide the basis for the implementation of holistic and appropriate WHM, it appears that the care facilities of all settings worked together with external cooperation partners concerning WHP (Figure 1). Regarding the “*employee participation in WHP*”, home-based LTC achieved 100%, whereas LTC facilities only achieved 71%. Lower results were also visible for 71% of LTC facilities having installed a “*steering group for WHP*”, whereas 100% of the acute care hospitals confirmed this. The lowest results for overall items and settings for WHP were achieved for acute care hospitals seeing “*WHP as a part of leadership-training*” (25%).

**Figure 2:**
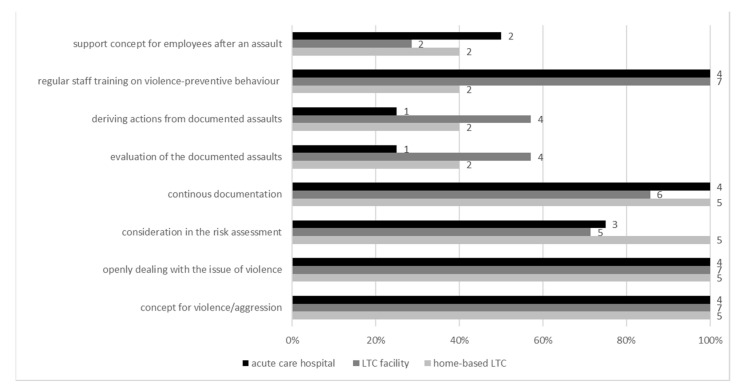
Existence of structures and processes for handling incidents of violence and aggression (per set-ting).

Since violence is also a burden on health, the survey asked about structures for preventing and dealing with violence. Across all settings, the highest proportion (100%) of *handling of incidents of violence and aggression* was found in the items “*openly dealing with the issue of violence*” and “*concept for violence/aggression*”. The results for “*deriving actions from documented assaults*” and “*evaluation of the documented assaults*” were the lowest in all settings, especially for acute care hospitals. Additionally low, but slightly higher rated for acute care hospitals was “s*upport concept for employees*” (Figure 2).

**Figure 3:**
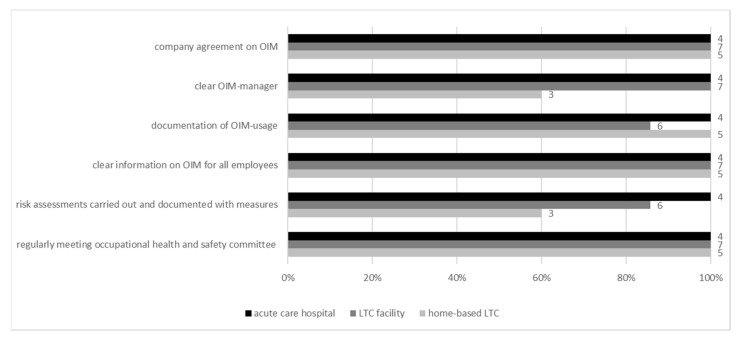
Existence of structures and processes for *operational integration management* (OIM) and occupational safety (per setting).

OIM—as one of the three columns of WHM—was nearly always fully established. In all settings, there was a “*company agreement on OIM”* and “*clear information on OIM for all employees*” about this offer. In total, 40% of the outpatient care services did not have clear information on who the “*OIM-manager*” was (Figure 3). Concerning *occupational safety* as the third column of WHM, it became apparent that a “*regularly meeting occupational health and safety committee*” was established across all settings. All acute hospitals seemed to carry out the “*risk assessment”* and its documentation, compared with only 60% of outpatient care service facilities (Figure 3).

## 4. Discussion

### 4.1. Summary of Findings

The objective of this study was to examine differences in WHM structures and processes in three distinct settings of nursing. Even though the small number of cases in this explorative study did not reveal statistically significant differences, home-based LTC showed the best results on organizational framework conditions for WHM for nearly all indexes assessed.

Overall, all structures measured here can be rated as quite well developed across all settings. This also could fit in with the majority’s good assessment of the result of *social capital* (descriptive characteristic of the atmosphere in the care facility). Nevertheless, the need for action to close the gap to a holistic and sustainable WHM has become clear in certain points. This is especially the case for the implementation of WHM as a systematic management process, a support concept for violence assaults and its evaluation. Furthermore, progress in terms of WHP being part of leadership training and balanced WPH activities targeting the behavioral-related and environmental-related level would make sense. The two remaining columns to fulfill WHM—*operational integration management* and *occupationally safety*—were nearly fully established across all settings (which is mandatory in Germany).

### 4.2. Study Aims in Context

Our study revealed that all three nursing settings showed better results in *health promoting willingness* compared to studies with small- and medium-sized enterprises (SMEs) [26] and information and communication technology (ICT) companies in Germany [27]. Indeed, the same applies to *health promotion management* [26]. This could be due to a fundamentally stronger interest of the care facilities in WHP, which also motivated the care facilities in our study to participate in a WHM project. Care facilities with a lower interest in WHM might have not been interested in participating in a WHM project.

The *health promoting willingness* of a company to implement WHM on a permanent basis [26] was the best rated index for the settings of home-based LTC and LTC facilities in our study. However, there was a decline in the *health promoting willingness* for WHP to the concrete existing *health promotion management* structures in these two settings. For acute care hospitals, the gap was displayed vice versa, which may result from the lowest results for *health promoting willingness* but the best results for *health promotion management*. Acute care hospitals seem to have installed more structures but show a weaker will than LTC facilities and home-based LTC.

As the *health promotion management index* represents many criteria that are considered important for WHM, a lack of recommended structures for WHM—e.g., a structured plan [20], process monitoring and evaluation [20,21] and the provision of personnel and financial resources—in the overall settings could be possible [22,23]. Especially for LTC facilities, there is at least one facility without any *health promoting management* structures at all, whereas most facilities show a medium existence of recommended structures.

In terms of *social capital*, our acute care hospitals showed lower results (0.7 point) in European comparison (mean = 2.6). A survey of hospital managers on *social capital* conducted across Europe in 2013 showed better results, with an average rating of 3.3 (range 1–4) [32]. This European study displayed that higher levels of *social capital* are associated with higher implementation of measures to improve quality, which is an interesting topic in terms of operational framework conditions for employee health [32]. A reason for lower results in our study could be the ongoing COVID-19 pandemic situation being a burden for acute care hospitals. Existing structural deficits in the area of nursing became more clearly visible as a result [33]. Comparing our findings in the same sector, study results on home-based LTC (*n* = 176) in northern Germany are in line with our findings on the implementation of a *workplace health activity index* [34]. As our results for the *workplace health activity index* in acute care hospitals were below the LTC care facilities, we cannot confirm other findings, suggesting that WHP offers are predominantly available in large facilities such as hospitals and not often in medium-sized LTC facilities [35,36]. This could be related to the fact that most of our participating LTC facilities are comparably large and have 50–249 employees. This might enable similarly good organizational conditions or networking with cooperation partners to offer a wide range of WHP activities, as it is usual in larger companies such as hospitals. However, it could also be possible that the *health promoting willingness*—which is rated quite highly in our results—promotes the implementation of more activities in our care facilities.

Due to the high prevalence of violence, it also holds strong importance to create structures for this purpose. This appears to be quite good at our facilities, but it could also be a distortion of the overall situation, as another study shows. Two-thirds of the employees in healthcare facilities (63.8%, *n* = 1984) who have experienced violence did not feel well prepared by the institution for such attacks [6]. Our results showed a better situation for having a concept of violence/aggression and training staff accordingly. Nevertheless, there is still a need for evaluation and deriving actions from documented assaults across all settings. Whereas our results for the structure of regularly meeting occupational health and safety committee was rated at 100% for home-based LTC, a German study from 2016 to 2019 displayed that only 22% of the companies had a management system with integrated occupational health and safety. Overall occupational health and safety organization, was rated suitable in 49%. In our study 60% of the home-based LTC carried out an appropriate risk assessment, whereas in the nationwide study it was only 38%. For LTC facilities in the nationwide survey, one-third have a management system with integrated occupational health and safety while our results showed this was the case for 100%. Overall, occupational health and safety organization was rated suitable in 70% of the nationwide LTC facilities. In our study 86% of the LTC facilities carried out an appropriate risk assessment, whereas in the nationwide study it was only 56%. Whereas our results for the structure of regularly meeting occupational health and safety committee met by 100% for acute care hospitals, a German study from 2016 to 2019 displayed that only 40% of the companies had a management system with integrated occupational health and safety. Overall occupational health and safety organization was rated suitable in 88%. In our study, 100% of the home-based LTC carried out an appropriate risk assessment, whereas in the nationwide study it was only 73% [37]. Again the reason for this overall better result may be found in a general higher interest in WHM, as the companies decided to participate in a workplace health related topic. Since there is a lack of setting-specific comparisons for OIM in the literature, the results presented here are compared with a nationwide, cross-sector survey. In 2007, 28% of small companies, 38% of medium-sized companies and 68% of large companies had already implemented or carried out OIM. Our items on the existence of a company agreement on OIM, a clear OIM-manager, documentation of OIM usage and information on OIM were all at least 60%, with the emphasis on 100% compliance. In the future, it would make sense to conduct more setting-specific and company size-specific surveys on OIM [38].

Given the high level of incapacity to work and the demographic development, it is clear that solutions must be found to keep nurses healthy and on the job. WHM can contribute to improved workability and its necessary is underlined by the fact that 75% of nurses are actually interested in WHM [5]. The fact that challenges still exist in this regard is shown by our results, among other things, by the gap between the will and the actually implemented systematic WHM process. A similar picture emerges in other care facilities where often only superficial or exclusively behavioral preventive measures for health are implemented [7]. Challenges in the holistic implementation of WHM are also shown by American analyses naming difficulties such as a lack of management support, a lack of qualified providers, a lack of qualified staff and emerging costs. Other challenges included a lack of space and a lack of interest among employees [39]. However, good workplace health management always requires work at the behavioral-related level as well as the environmental-related level embedded in suitable WHM structures [11]. For example, the strong influence of digitalization tends to shift responsibility for (workplace) health to the individual through digital personal offerings [40].

In summary, it can be said that there is both a need and a necessity to promote holistic WHM. Therefore, as examined in this study, a structured plan for WHM [20], process monitoring, evaluation [21], the provision of adequate resources in terms of personnel and finances [23,24], steering groups/similar recurring meetings [24,25], networking of internal and external actors [19], systematic integration of WHP activities, occupational integration management and occupational safety [26] are necessary for WHM.

### 4.3. Limitations

Although there were several noteworthy aspects to this study, our study also has several limitations. First, the small sample size should be mentioned in this regard, which means that it is not possible to generalize the results for the German care sector. Results can only be seen in relation to the subpopulation from which the sample is drawn [41]. However, our development of a WHM check as part of the BAGGer project is valuable preliminary work for a representative survey in further studies. We would like the WHM-check to be carried out in the future with a larger sample of nursing settings as well as other company branches in order to obtain generalizable and comparative results and a higher external validity. Second, when discussing the questionnaire, it was communicated that many WHM activities had recently been suspended due to the COVID-19 pandemic, so it can be assumed that WHM was better positioned in parts before the pandemic. The still relatively high values achieved by the project care facilities just at the beginning of a project in the parameters presented here could be due to the fact that the project companies have a fundamentally stronger interest in WHM and therefore participated in a WHM project.

The question that arises is whether WHM structures and processes that are assessed as “good” by the care facility manager are also perceived as “good” WHM by the nurses.

The results presented here have revealed insights into the state of workplace health management structures from a management perspective. The decision to interview these managers with personnel responsibility for nurses was based on the idea that they have the most knowledge about structures and processes of the care institution. The assessed items may not be directly visible to the employees, for example topics such as financial resources or workplace health being part of leadership-training. They rather address topics that are relevant for the management of WHM. However, distortions could have arisen due to social desirability in the answers by the care facility manager. There is a risk that the extent to which WHM activities are actually perceived by employees will be overlooked. A survey that assesses to what extent WHM structures and activities are recognized by employees would be useful to obtain an actual and multi-perspective status of how WHM is perceived.

Third, there is a fundamental lack of scientifically tested instruments that measure German WHM structures across the three columns of WHP, occupational safety and OIM. Particularly in the area of violence prevention, OIM and occupational safety, there is a need for further research to provide quantitative measurement instruments that can be used in Germany. The categorization of *health promotion capacity* was only possible here with reservations [26,27]. The latent constructs were the same, but Jung et al. used partly different items after having adjusted them with factor loading [27]. The use of closed questions due to the questionnaire sources allows a numerical comparison of the results. However, with the target group of managers, a format with open questions would be interesting, which would address the complexity of the subject matter.

This survey has made it possible to establish a status quo of factors as well as differences in the areas of WHP, OIM and occupational safety in Germany and thus also identified areas where action is needed.

## 5. Conclusions

There is an evidence-based need for action for holistic WHM in the nursing sector due to the high workload, high sickness rates and strong fluctuation in leaving the nursing profession [7]. The results for WHM in the care facilities represented here seem to be better than average, although there is still work to be done to improve those management structures and processes in the dimensions that achieved weaker results. This would close the gaps between only being willing to promote workplace health and actually building up the organizational framework conditions for sustainable WHM. In practice, the focus should be on improving the management process for WHM, particularly the allocation of human, financial and time resources, as well as the analysis, goal setting and evaluation and provision of information on WHM. Moreover, workplace health activities should be implemented which target the environmental- and individual-related level. Furthermore, it is recommended to make WHM a leadership topic, while a need for action also lies in implementing a support concept for violence assaults, evaluating documented assaults and deriving suitable actions.

There is still further research needed in ascertaining what is significant for promoting WHM in care facilities. For the practical implementation, it would be helpful to explore which promotion factors and barriers in the WHM in care facilities can be expected and worked on. Further, it would be interesting to compare the results presented here with an employee survey examining workability, health status, violence experiences and subjective *social capital* to examine correlations between organizational framework conditions and health-related outcomes. Seeing results from a baseline as well as a follow-up study would be an enrichment. The comparison even with a third data resource displaying company key figures such as absenteeism, fluctuation, OIM quotas, applicant quotas could enrich such a calculation and provide indications of interrelationships.

As the literature draws out a lack of important considered environmental-related health promotion issues, more studies on this topic would be helpful. An interesting question would be to explore the distribution of environmental- and individual-related WHM in a larger sample of companies and the connection to the health status of the employees. Furthermore, investigations into the effect of environmental-related WHM would be purposeful.

Overall, it can be said that through this survey it was possible to measure the status quo of selected organizational frameworks for WHM in different settings of nursing that are considered recommendable in the literature. Such a survey can provide interesting results both as a baseline and follow-up measurement and can function as a comparison of several companies or settings.

## Figures and Tables

**Table 1:** Overview of the sample composition.

Care Facilities	Home-Based LTC (5; 31%)	LTC Facility (7; 44%)	Acute Care Hospital (4; 25%)
Number of all employees (nurses and others)(mean, (± SD), [min–max], (Med))	146 (±153) [16–326], (49)	91 (±27) [56–131], (93)	532 (±415) [158–1125] (422)
Company size (number of all employees; nurses and others)			
<10	0	0	0
10–49	3	0	0
50–249	0	7	1
>250	2	0	3

**Table 2:** Overview of the questionnaires used for workplace health promoting structures and processes (WHM-check).

Main Topic	Questionnaire	Response Option
Health promoting capacity [26,27]	*Health promoting willingness* *index* over three items [28]	Eleven-point Likert scale (from 0 = “do not agree at all”, 10 = “fully agree”)
	*Health promoting management* *index* over nine items [28]	Eleven-point Likert scale (from 0 = “do not agree at all”, 10 = “fully agree”)
Social capital	*Social capital* *index* over nine items [28]	Four-point Likert scale (1 = “do not agree at all”, 4 = “fully agree”)
Workplace health promotion	*Workplace health activities* *index* over six items [28]	Eleven-point Likert scale (0 = “do not agree at all”, 10 = “fully agree”)
	*Workplace health structures* and supporting cooperation partner five items [28]	Four nominal scaled response options (“yes” and “no”) One non-standardized question (“work with cooperation partner”)
*Handling of incidents of violence* and aggression	*Handling of incidents of violence and aggression* (against employees) eight items [30]	Nominal scaled response options (“yes” and “no”)
*Operational integration management*	*Operational integration management* four items [31]	Nominal scaled response options (“yes” and “no”)
Occupational safety	Occupational safety two items non-standardized question	Nominal scaled response options (“yes” and “no”)

**Table 3:** Indexes per setting.

	Home-Based LTC (*n* = 5)	LTC Facilities (*n* = 7)	Acute Care Hospital (*n* = 4)	*p* *
*Health promoting willingness index* (mean(±SD), [min:1–max:10]; (Med))	7.4 (±1.2) [6–9]; (7.0)	6.8 (±1.8) [4–9]; (7.0)	5.9 (±1.2) [4–7]; (6.0)	*p* = 0.368
*Health promoting management index* (mean(±SD), [min:1–max:10]; (Med))	6.2 (±2.1) [4–9]; (5.8)	5.5 (±2.9) [0–9]; (5.3)	6.3 (±2.0) [5–9]; (5.6)	*p* = 0.831
*Social capital index* (mean(±SD), [min:1–max:4]; (Med))	3.3 (±0.2) [3.0–3.8]; (3.4)	3.1 (±0.4) [2.6–3.7]; (3.2)	2.6 (±0.3) [2.3–3.9]; (2.7)	*p* = 0.015
*Workplace health* *activities index* (mean(±SD), [min:1–max:10]; (Med))	6.3 (±2.6) [3–9]; (6.0)	5.5 (±2.1) [2–9]; (6.0)	5.1 (±1.0) [4–7]; (4.8)	*p* = 0.712

* univariate analysis of variance.

## Data Availability

The data presented in this study are available on request from the corresponding author for valid reasons. The data are not publicly available due to privacy reasons of the participating care facilities.

## Supplementary Materials

The following supporting information can be downloaded at: https://www.mdpi.com/article/10.3390/ijerph19063693/s1, Table S1: Detailed overview of the questionnaires and items used for the WHM-check [27,28,30,31].

## References

[1] Slotala L., Jacobs K., Kuhlmey A., Greß S., Klauber J., Schwinger A. (2020). Stellschrauben mit großer Wirkung. Pflege-Report 2019: Mehr Personal in der Langzeitpflege-Aber Woher?.

[2] Schwinger A., Klauber J., Tsiasioti C., Jacobs K., Kuhlmey A., Greß S., Klauber J., Schwinger A. (2020). Pflegepersonal heute und morgen. Pflege-Report 2019: Mehr Personal in der Langzeitpflege-Aber Woher?.

[3] Sell L., Bültmann U., Rugulies R., Villadsen E., Faber A., Søgaard K. (2009). Predicting long-term sickness absence and early retirement pension from self-reported work ability. Int. Arch. Occup. Environ. Health.

[4] Drupp M., Meyer M., Jacobs K., Kuhlmey A., Greß S., Klauber J., Schwinger A. (2020). Belast ungen und Arbeitsbedingungen bei Pflegeberufen–Arbeitsunfähigkeitsdaten und ihre Nutzung im Rahmen eines Betrieblichen Gesundheitsmanagements. Pflege-Report 2019: Mehr Personal in der Langzeitpflege-Aber Woher?.

[5] Ehegartner V., Kirschneck M., Frisch D., Schuh A., Kus S. (2020). Arbeitsfähigkeit von Pflegekräften in Deutschland–welchen Präventionsbedarf hat das Pflegepersonal: Ergebnisse einer Expertenbefragung. Gesundheitswesen.

[6] Schablon A., Wendeler D., Kozak A., Nienhaus A., Steinke S. (2018). Prevalence and Consequences of Aggression and Violence towards Nursing and Care Staff in Germany—A Survey. Int. J. Environ. Res. Public Health.

[7] Krupp E., Hielscher V., Kirchen-Peters S., Jacobs K., Kuhlmey A., Greß S., Klauber J., Schwinger A. (2020). Betriebliche Gesundheitsförderung in der Pflege–Umsetzungsbarrieren und Handlungsansätze. Pflege-Report 2019: Mehr Personal in der Langzeitpflege-Aber Woher?.

[8] Barthelmes I.W., Bödeker J., Sörensen K.-M., Kleinlercher J.O. (2019). Iga.Report 40. Wirksamkeit und Nutzen Arbeitsweltbezogener Gesundheitsförderung und Prävention.: Zusammenstellung der Wissenschaftlichen Evidenz 2012-2018.

[9] Pfannstiel M.A., Mehlich H. (2018). BGM–Ein Erfolgsfaktor für Unternehmen.

[10] Elke G., Gurt J., Möltner H., Externbrink K. (2015). Arbeitsschutz und Betriebliche Gesundheitsförderung–Vergleichende Analyse der Prädiktoren und Moderatoren Guter Praxis.

[11] Bauer S., Geiger L., Niggemann R., Seidel J. (2020). Medizinischer Dienst des Spitzenverbandes Bund der Krankenkassen e. V. (MDS. PRÄVENTIONSBERICHT 2020: Leistungen der gesetzlichen Krankenversicherung:Primärprävention und Gesundheitsförderung. Leistungen der sozialen Pflegeversicherung:Prävention in stationären Pflegeeinrichtungen. Berichtsjahr 2019. https://md-bund.de/fileadmin/dokumente/Publikationen/GKV/Praevention/2020/Praeventionsbericht_2020_barrierefrei.pdf.

[12] Badura B., Ritter W., Scherf M. (1999). Betriebliches Gesundheitsmanagement-Ein Leitfaden Für Die Praxis.

[13] Hoek R.J.A., Havermans B.M., Houtman I.L.D., Brouwers E.P.M., Heerkens Y.F., Zijlstra-Vlasveld M.C., Anema J.R., van der Beek A.J., Boot C.R.L. (2017). Stress Prevention@Work: A study protocol for the evaluation of a multifaceted integral stress prevention strategy to prevent employee stress in a healthcare organization: A cluster controlled trial. BMC Public Health.

[14] Wijnen B.F.M., Lokkerbol J., Boot C., Havermans B.M., van der Beek A.J., Smit F. (2020). Implementing interventions to reduce work-related stress among health-care workers: An investment appraisal from the employer's perspective. Int. Arch. Occup. Environ. Health.

[15] Olson R., Wright R.R., Elliot D.L., Hess J.A., Thompson S., Buckmaster A., Luther K., Wipfli B. (2015). The COMPASS pilot study: A total worker Health™ intervention for home care workers. J. Occup. Environ. Med..

[16] Nejati A., Shepley M., Rodiek S., Lee C., Varni J. (2016). Restorative Design Features for Hospital Staff Break Areas: A Multi-Method Study. HERD Health Environ. Res. Des. J..

[17] Cho H., Han K. (2018). Associations Among Nursing Work Environment and Health-Promoting Behaviors of Nurses and Nursing Performance Quality: A Multilevel Modeling Approach. J. Nurs. Scholarsh..

[18] Kleina T., Brause M., Horn A. (2013). Potenziale und Probleme der Gesundheitsförderung bei Pflegepersonal in stationären Pflegeeinrichtungen. Gesundheitswesen.

[19] GKV-Spitzenverband Leitfaden Prävention: Handlungsfelder und Kriterien nach §20 Abs.2 SGBV: Berlin, Germany, 2020. http://www.bdem.de/pdf/Leitfaden-Praevention.pdf.

[20] Holden R.J., Or C.K.L., Alper S.J., Joy Rivera A., Karsh B.-T. (2008). A change management framework for macroergonomic field research. Appl. Ergon..

[21] Helfrich C.D., Damschroder L.J., Hagedorn H.J., Daggett G.S., Sahay A., Ritchie M., Damush T., Guihan M., Ullrich P.M., Stetler C.B. (2010). A critical synthesis of literature on the promoting action on research implementation in health services (PARIHS) framework. Implement. Sci..

[22] Lee S.-Y.D., Weiner B.J., Harrison M.I., Belden C.M. (2013). Organizational transformation: A systematic review of empirical research in health care and other industries. Med. Care Res. Rev..

[23] Semenic S., Childerhose J.E., Lauzière J., Groleau D. (2012). Barriers, facilitators, and recommendations related to implementing the Baby-Friendly Initiative (BFI): An integrative review. J. Hum. Lact..

[24] Kotter J.P. (2008). Change Management-Das Unternehmen erfolgreich erneuern. Harvard-Business-Manager: Das Wissen der Besten.

[25] (1988). Sozialgesetzbuch Fünftes Buch—Gesetzliche Krankenversicherung, § 20f Landesrahmenvereinbarungen zur Umsetzung der nationalen Präventionsstrategie: §20f SGB V.

[26] Biallas B., Schäfer D., Dejonghe L., Franz L., Petrowski K., Froböse I., Wilke C. (2019). Präventionsreife in kleinen und mittleren Unternehmen. Prävention Und Gesundh..

[27] Jung J., Nitzsche A., Neumann M., Wirtz M., Kowalski C., Wasem J., Stieler-Lorenz B., Pfaff H. (2010). The Worksite Health Promotion Capacity Instrument (WHPCI): Development, validation and approaches for determining companies' levels of health promotion capacity. BMC Public Health.

[28] Pfaff H., Nitzsche A., Jung J. (2008). Handbuch zum Healthy Organisational Resources and Strategies. (HORST) Fragebogen: Forschungsbericht 03-2008.

[29] Bourdieu P., Bauer U., Bittlingmayer U.H., Scherr A. (2012). Ökonomisches Kapital, kulturelles Kapital, soziales Kapital. Handbuch Bildungs-und Erziehungssoziologie.

[30] Berufsgenossenschaft für Gesundheitsdienst und Wohlfahrtspflege Selbsteinschätzung Gewalt und Aggression. https://www.bgw-online.de/SharedDocs/Downloads/DE/Arbeitssicherheit_und_Gesundheitsschutz/Organisationsberatung/Selbsteinschaetzung_Download.pdf?__blob=publicationFile.

[31] Deutsche Rentenversicherung Baden-Württemberg Praxisleitfaden für Kleine und Mittelständische Unternehmen. https://kom-consulting.de/pages/BEM_Broschuere_DRV-BW-2014.pdf.

[32] Hammer A., Pfaff A., Hammer A., Pfaff H. (2013). Der Einfluss von Sozialkapital im Krankenhausmanagement auf die Implementierung von Qualitätsmanagementsystemen in europäischen Krankenhäusern. German Medical Science GMS Publishing House.

[33] Jacobs K., Kuhlmey A., Greß S., Klauber J., Schwinger A. (2020). Pflege-Report 2019: Mehr Personal in der Langzeitpflege-Aber Woher?.

[34] Koinig I., Diehl S. (2021). Healthy Leadership and Workplace Health Promotion as a Pre-Requisite for Organizational Health. Int. J. Environ. Res. Public Health.

[35] BKK Dachverband (2017). Gesundheit und Arbeit-Blickpunkt Gesundheitswesen: BKK Gesundheitsatlas 2017.

[36] Stier-Jarmer M., Frisch D., Oberhauser C., Berberich G., Schuh A. (2016). The Effectiveness of a Stress Reduction and Burnout Prevention Program. Dtsch. Arztebl. Int..

[37] Liese A., Smieszkol C., Wittreck H. (2013). Sicherheit und Gesundheitsschutzbei der Pflege: Abschlussbericht zum GDA-Arbeitsprogramm.

[38] Niehaus M., Marfels B., Vater G., Magin J., Werkstetter E. Betriebliches Eingliederungsmanagement: Studie zur Umsetzung des Betrieblichen Eingliederungsmanagements nach § 84 Abs. 2 SGB IX: (Forschungsbericht /Bundesministerium für Arbeit und Soziales, FB374). https://nbn-resolving.org/urn:nbn:de:0168-ssoar-265779.

[39] Weinstein M., Cheddie K. (2021). Adoption and Implementation Barriers for Worksite Health Programs in the United States. Int. J. Environ. Res. Public Health.

[40] Faller G. (2020). Wearable und App: Was kommt auf uns zu?. Gute Arb..

[41] Andrade C. (2021). The Inconvenient Truth About Convenience and Purposive Samples. Indian J. Psychol. Med..

