# Supplementary material for: Organizational Framework Conditions for Workplace Health Management in Different Settings of Nursing—A Cross-Sectional Analysis in Germany
Source: Int J Environ Res Public Health. 2022 Mar 20;19(6):3693. doi: 10.3390/ijerph19063693 (PMC8952851; doi:10.3390/ijerph19063693)
Supplement: Supplementary file 1 [file ijerph-19-03693-s001.zip › ijerph-1602384-supplementary.pdf]

Table S1: Detailled overview of the questionnaires and items used for the WHM-check

Questionnaire	Items	Response option
**Health promoting willingness[28]**<br>index over three items	In our company, the prevailing opinion is that health is exclusively a personal matter.[1]<br><br>In our company, the subject of employee health promotion is often discussed. [1]<br><br>The company's management demonstrates a very strong willingness to actively promote employee health. [1]	Eleven-point Likert scale (from 0 = "do not agree at all", 10 = "fully agree")
**Health promoting management[28]**<br>index over nine items	Time resources, such as time off for employees, are made available for workplace health promotion on a large scale. [2]<br><br>Financial resources for workplace health promotion are provided on a large scale. [2]<br><br>Large amounts of personnel resources, e.g., through specially created jobs, are made available for workplace health promotion. [2]<br><br>Needs are systematically identified prior to the implementation of worksite health promotion measures. [1]<br><br>We define quantifiable goals for worksite health promotion. [1]<br><br>All worksite health promotion measures are constantly evaluated based on the defined goals. [1]<br><br>The worksite health promotion measures are modified based on evaluation results.. [1]<br><br>All employees are informed about the projects in the area of workplace health promotion by suitable means of internal public relations work. [2]<br><br>Our company has a comprehensive health promotion program, in which individual measures are integrated. [1]	Eleven-point Likert scale (from 0 = "do not agree at all", 10 = "fully agree")

**Social capital[28]** index over nine items	Now that you think about collaboration and cooperation in your facility, how strongly do you agree with the following statements?<br><br>...there is unity and agreement.<br><br>...how much do we trust each other.<br><br>... there is a ""we feeling"" among the employees.<br><br>... the working atmosphere is good.<br><br>...we get along poorly with each other.<br><br>...there is a great willingness to help each other.<br><br>...we share many values.<br><br>...there is a relationship of trust between the hierarchy levels.<br><br>...there is respect between the members of the different hierarchy levels. [2]	Four-point Likert scale (1=”do not agree at all”, 4=”fully agree”)
**Workplace health activities [28]** index over six items	Our company offers a wide range of measures to change employees' health behavior (e.g. smoking prevention). [2]<br><br>Our company offers a wide range of preventive measures (e.g. on cancer). [2]<br><br>Our company improves the working conditions of employees through a variety of measures (e.g. measures to improve work organization). [2]<br><br>Our company offers a wide range of measures to promote and improve mental health (e.g. stress management measures). [2]<br><br>Our company offers a wide range of measures to promote the well-being of employees (e.g., appealing rooms, physiotherapeutic measures). [2]	Eleven-point Likert scale (0 = “do not agree at all”, 10 = “fully agree”)

	Our company offers a wide range of measures to help employees do their work full of energy (e.g. relaxation measures). [2]	
**Workplace health structures [28] and supporting cooperation partner**<br>five items	There is a steering committee or project group for workplace health promotion. [2]<br><br>Workplace health promotion is part of the training and continuing education of managers. [2]<br><br>All employees have the opportunity to actively participate in the design and planning of workplace health promotion. [2]<br><br>Workplace health promotion is the responsibility of the individual company units.[1] [2]	Four nominal scaled response options (“yes” and “no”)
	We work together with cooperation partners (e.g. social insurance carriers) to promote WHP. [2]	non-standardized question (“work with cooperation partner”)
**Handling of incidents of violence and aggression (against employees) [30]**<br>eight items	Our organization has a written concept (or a strategy paper, a guideline, a service agreement ...) for dealing professionally with violence and aggression. [2]<br><br>In our organization, the topic of "violence and aggression" is dealt with in an open, objective and constructive manner. [2]<br><br>Hazards due to incidents of violence and aggressive behavior are addressed in the risk assessment. [2]<br><br>Violent and aggressive incidents are continuously documented in our organization (e.g., as accident reports, in the first-aid book, as file notes). [2]<br><br>A regular evaluation of the documented violent assaults is carried out (e.g. according to the number of incidents per area, according to professional experience and gender of the persons concerned). [2]<br><br>Conclusions that guide action are drawn from the regular evaluation of documented incidents of violence (e.g., revision of company emergency plans, training of managers). [2]	Nominal scaled response options (“yes” and “no”)

	Our organization regularly offers specific training on violence-preventing behavior for employees (e.g., de-escalation training). [2]	
	There is a support concept for employees who have been exposed to a violent event. [2]	
**Operational integration management [31]** four items	Are all employees aware of the possibility of an OIM? [2]	Nominal scaled response options (“yes” and “no”)
	Is the answer, whether participation in the OIM or not, documented? [2]	
	Has it been clarified who can assume the function of an OIM representative? [2]	
	Is there a written schedule or a company agreement on the OIM? [2]	
**Occupational safety** two items non-standardized question	Is there an occupational health and safety committee that meets regularly (e.g. as part of the WHM steering committee) or a person responsible for occupational health and safety management? [2]	Nominal scaled response options (“yes” and “no”)
	Have the risk assessments required by the German Ordinance on Industrial Safety and Health (BSV) been carried out and documented with measures? [2]	

[1] Translation from Jung et al., 2010 [27]

[2] Own translations. These do not constitute a validated English-language questionnaire and are for informational purposes only.

Reference

[27] Jung, J., Nitzsche, A., Neumann, M., Wirtz, M., Kowalski, C., et al., “The Worksite Health Promotion Capacity Instrument (WHPCI): development, validation and approaches for determining companies' levels of health promotion capacity,” *BMC public health*, Vol. 10, 1 Jan. 2010, p. 550. doi: 10.1186/1471-2458-10-550.

[28] Pfaff, H., Nitzsche, A., and Jung, J., *Handbuch zum „Healthy Organisational Resources and Strategies“. (HORST) Fragebogen. Forschungsbericht 03-2008*, 1 Jan. 2008.

[30] Berufsgenossenschaft für Gesundheitsdienst und Wohlfahrtspflege, “Selbsteinschätzung „Gewalt und Aggression,” URL: https://www.bgw-online.de/SharedDocs/Downloads/DE/Arbeitssicherheit_und_Gesundheitsschutz/Organisationsberatung/Selbsteinschaetzung_Download.pdf?__blob=publicationFile [retrieved 14 December 2021].

[31] Deutsche Rentenversicherung Baden-Württemberg, “Praxisleitfaden für kleine und mittelständische Unternehmen,” URL: https://kom-consulting.de/pages/BEM_Broschuere_DRV-BW-2014.pdf [retrieved 8 March 2021].
